# Epidermal growth factor receptor is expressed and active in a subset of acute myeloid leukemia

**DOI:** 10.1186/s13045-016-0294-x

**Published:** 2016-08-03

**Authors:** Hasan Mahmud, Steven M. Kornblau, Arja ter Elst, Frank J. G. Scherpen, Yi Hua Qiu, Kevin R. Coombes, Eveline S. J. M. de Bont

**Affiliations:** 1 Department of Pediatrics, Division of Pediatric Oncology/Hematology, Beatrix Children’s Hospital, University Medical Center Groningen, University of Groningen, PO Box 30.001, 9700 RB Groningen, The Netherlands; 2 Department of Stem Cell Transplantation and Cellular Therapy, MD Anderson Cancer Center, University of Texas, Houston, TX USA; 3 Department of Biomedical Informatics, Wexner Medical Center, The Ohio State University, Columbus, OH USA

**Keywords:** EGFR, AML, RPPA, Kinome, Leukemia

## Abstract

The epidermal growth factor receptor (EGFR) inhibitor erlotinib has been shown to induce complete remission of acute myeloid leukemia (AML) in two patients with concurrent lung cancer and raised attention for a role of EGFR in AML whereas a recent phase II clinical study with gefitinib in AML demonstrated a negative result on the outcome. However, from several studies, EGFR expression in AML is poorly defined and the role of EGFR in AML remains unclear. Herein, we report the results of EGFR expression in AML of large cohorts of adult and pediatric AML patients with the data of total protein and phosphorylation levels of EGFR. Our data conclude that there is the expression of EGFR at the protein level in a subset of AML, which was identified to be functionally active in ~15 % of AML patients. This suggests that future studies need to be conducted with a subset of AML patients characterized by high EGFR expression.

**Electronic supplementary material:**

The online version of this article (doi:10.1186/s13045-016-0294-x) contains supplementary material, which is available to authorized users.

Epidermal growth factor receptor (EGFR) expression in acute myeloid leukemia (AML) cells is a subject of controversy, as there is no consensus about the expression and activity of EGFR in AML. In non-small cell lung cancer (NSCLC) patients, EGFR is known to be highly expressed. The EGFR inhibitor erlotinib was shown to induce complete remission of AML in two adult patients with concurrent NSCLC and raised attention for EGFR in AML [1, 2]. Especially NSCLC patients with rare EGFR mutations had lower response rates to EGFR inhibitors than the patients with common mutations [3], due to the counteraction of EGFR tyrosine kinase inhibitors (TKIs) with specific EGFR mutations. In AML, previous reports showed that erlotinib was able to induce in vitro differentiation, cell cycle arrest, and apoptosis of AML blasts [4]. Another study showed that gefitinib, another EGFR inhibitor, induced myeloid differentiation in AML [5]. Additionally, EGFR expression was confirmed by an experimental murine tumor of AML origin [6]. Gene expression of larger adult and pediatric AML samples detected EGFR expression previously [7, 8]. In contrast, EGFR protein levels, as assessed by immunochemistry, and mRNA levels of EGFR have been found to be doubtfully low in AML blasts [9, 10]. In AML cell lines, EGFR is not expressed both at protein and mRNA levels and the phenotypic effects of the EGFR inhibitors must be due to off-target effects [9, 11]. Recently, Deangelo et al. investigated the effect of EGFR inhibitor gefitinib in adult AML patients (*n* = 18) in a phase II clinical study [12]. Their results suggested that the single-agent gefitinib has no effect on patient outcome due to undetectable EGFR expression levels, both mRNA and protein. Therefore, data on whether EGFR is expressed, the actual level of expression, and if EGFR is present in an activated post-translationally modified phosphorylated state in AML has not been consistent in previous studies using small subsets of AML patients. Herein, we demonstrate the EGFR expression in total protein and protein phosphorylation levels in a well-defined subset of patients in large cohorts of AML patients.

We investigated total EGFR protein expression as well as EGFR phosphorylation in AML blasts using reverse phase protein array (RPPA) in a large cohort of adult AML patients (*n* = 511) and EGFR peptide phosphorylation levels using peptide phosphorylation array of AML patients both pediatric and adult (*n* = 96 + 83 = 179). The details of the sample collections and the methods of RPPA and peptide phosphorylation array are described [13, 14] in the design and methods section (Additional file 1: Design and methods). In this report, we demonstrate that the EGFR protein (*n* = 511) is expressed and active in a subset of AML patients. Expression of both total and Y992 phosphorylated EGFR protein was readily detected both in normal bone marrow (NBM)-derived CD34+ cells and in AML blasts, with expression following a Gaussian distribution. In the primary AML samples, expression of total EGFR protein was higher than that of NBM CD34+ cells in 11 % and expression of phosphorylated EGFR exceeded NBM in 18 % of cases (Fig. 1a, b). Interestingly, the total EGFR expression and EGFR phosphorylation data are correlated significantly (*p* < 0.0001). There was no significant difference of molecular and clinical characteristics (e.g., age, sex, WHO classification, FAB classification, karyotypes, blast percentage, white blood cell count, hemoglobin concentration, platelet count, complete response rate, relapse frequencies, death frequencies, FLT3-ITD, FLT3-D835, and NPM1 mutations) found between the 11 % AML (high EGFR) patients and the rest of the 89 % AML (low EGFR) patients (Additional file 2: Table S1). In addition, EGFR tyrosine kinase is functionally active in AML blasts, as demonstrated by peptide phosphorylation activity of EGFR-related peptides using peptide phosphorylation profiling arrays in a large cohort of AML patients (*n* = 179) (Fig. 1c). These results indicate that EGFR protein is both expressed and present in activated phosphorylated forms in AML, supporting EGFR as a potential therapeutic target in EGFR-expressing AML patients. The RPPA dataset is available at http://bioinformatics.mdanderson.org/supplements.html (under “RPPA Data in AML”) and the processed raw data of peptides phosphorylation can be found in the additional information (Additional file 3: Peptide phosphorylation data for Fig. 1c).

**Fig. 1:**
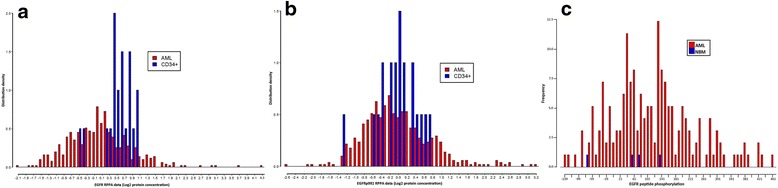
The total EGFR protein expression, phosphorylation of EGFR and EGFR peptides phosphorylation in primary AML samples. **a** Total EGFR protein expression was measured using RPPA of 511 adult AML samples. Total EGFR protein was highly expressed in 11 % (57/511) of the adult AML samples versus the CD34+ normal bone marrow (NBM) samples. The distribution density of total EGFR expression was depicted in the figure. **b** Similarly, the EGFR protein phosphorylation levels were measured using RPPA of the same 511 adult AML samples. EGFR protein phosphorylation levels were 18 % (93/511) higher in adult AML patient samples compared to the CD34+ NBM samples. **c** The EGFR peptide phosphorylations were measured using PepChipTM Kinomics microarray system of 179 AML patients. The median of four EGFR peptides with different phosphorylation sites was used to make the graph

The discordance between the readily detectable EGFR protein levels observed in this study and the lack of EGFR expression seen in some other analyses, as well as the lack of clinical efficacy of EGFR inhibitors seen in prior clinical trials, can be explained with our data. Patients included in previous studies could belong to the group of 85 % of AML samples which showed the same levels of EGFR expression as normal CD34+ cells in our study. The inclusion of only patients with low expression might account for the lack of response to gefitinib in AML patients (*n* = 18) evaluated in the phase II study by Deangelo et al. [12].

Altogether, our data shows increased expression of EGFR at both the total protein (11 %) and protein phosphorylation (18 %) levels in a subset of AML patients compared to normal CD34+ samples. Results of future clinical studies of EGFR inhibitors might be improved if they are restricted to patients with highly expressed EGFR or active phosphorylated EGFR. Notably, mutations of EGFR were not observed in the TCGA analyses of AML, so consideration of mutation status is not required in AML as it is for lung cancer [15].

## Abbreviations

AML, acute myeloid leukemia; EGFR, epidermal growth factor receptor; NBM, normal bone marrow; NSCLC, non-small cell lung cancer; RPPA, reverse phase protein array; TKIs, tyrosine kinase inhibitors

## Additional files

Additional file 1: Design and methods. (DOCX 15 kb) Additional file 2: Patient characteristics. (DOCX 18 kb) Additional file 3: Peptide phosphorylation data for Fig. 1c. (XLSX 22 kb)
