# Supplementary material for: Epidermal growth factor receptor is expressed and active in a subset of acute myeloid leukemia
Source: J Hematol Oncol. 2016 Aug 3;9:64. doi: 10.1186/s13045-016-0294-x (PMC4971659; doi:10.1186/s13045-016-0294-x)
Supplement: Additional file 1: — Design and methods. (DOCX 15 kb) [file 13045_2016_294_MOESM1_ESM.docx]

**Supplemental file 1: Design and methods**

***AML patient samples***

After getting written informed consent, the mononuclear cell fraction (MNC) of bone marrow and peripheral blood from healthy controls, adult (n=511 for RPPA and n=83 for peptide phosphorylation array) and pediatric AML patients (n=96) was obtained and cryopreserved. For this study adult and pediatric samples were approved by Medical Ethical Committee of M.D. Anderson Cancer Center, University of Texas, USA and by the University Medical Center Groningen, The Netherlands respectively. Samples were thawed rapidly at 37°C and diluted in a 6 mL volume of newborn calf serum and prepared for RPPA and peptide phosphorylation profiling array as described previously [13,14].

***Reversed Phase Protein Array (RPPA)***

Proteomic profiling was performed using newly diagnosed adult AML (n=511) samples and CD34+ normal bone marrow samples (n=20) using RPPA, as described previously [13]. Briefly, patient samples were printed in five serial dilutions onto slides along with normalization and expression controls. Slides were probed with a comprehensive set of 232 validated primary antibodies including antibodies against total EGFR (Santa Cruz Biologicals, Catalog # SC-03, primary antibody dilution 1:500, secondary antibody dilution 1:20,000) and EGFR phosphorylated on tyrosine 992 (Cell Signaling, Catalog #2235, primary antibody dilution 1:50, secondary antibody dilution 1:15,000), thereafter a secondary antibody incubation step to amplify the signal followed by a stable dye (40) precipitation. The stained slides were analyzed using Microvigene software (Vigene Tech) to produce quantified data.

***Peptide phosphorylation profiling array***

The peptide phosphorylation profiles of pediatric AML samples (n=96), adult AML samples (n=83) and normal bone marrow samples (n=4) were determined using the PepChipTM Kinomics microarray system (Pepscan, Lelystad, The Netherlands) as described previously [14]. The peptide array contains 976 different kinase peptide-substrates, each spotted as triplicates. The protein-derived peptide sequences contain phosphorylation sites that can be used as substrates for kinases active in the samples. The assay readout is the net sum of phosphorylation at each peptide, whether acted on by one kinase, or several different kinases. The chips were exposed to a phospho-imager plate for 72 hours, and the density of the spots was measured and analyzed with array software (ScanAlyze, version 2.50, Eisen Software, <http://rana.lbl.gov/eisen>).
