# Supplementary material for: Epidermal growth factor receptor is expressed and active in a subset of acute myeloid leukemia
Source: J Hematol Oncol. 2016 Aug 3;9:64. doi: 10.1186/s13045-016-0294-x (PMC4971659; doi:10.1186/s13045-016-0294-x)
Supplement: Additional file 2: — Patient characteristics. (DOCX 18 kb) [file 13045_2016_294_MOESM2_ESM.docx]

**Supplementary table 1: Patient Characteristics**

| **Characteristics (n=511)** | **~11% (57/511)AML patients (High EGFR)** | **~89% (93/511)AML patients (Low EGFR)** | **p-value** |
| --- | --- | --- | --- |
| *Age (years)* | 60.6 | 62.7 | 0.31 |
| *Gender (%)*   - Male - Female | 64.9  35 | 55.9  44 | 0.19 |
| *WHO-classification (%)*   - AML with recurrent genetic abnormalities - Therapy related - Multilineage dysplasia - Not in other | 14  12.2  24.5  49.1 | 8.5  14.5  21.1  55.7 | 0.47 |
| *French-American-British classification (%)*   - M0 - M1 - M2 - M4 - M5 - M6 - M7 - RAEBT - unknown | 3.5  12.2  38.5  12.2  5.2  12.2  1.75  8.7  5.2 | 5.9  10.5  32.3  24  11  4.4  1.9  6.6  3 | 0.11 |
| *Cytogenetics (%)*   - *Favorable* - *Unfavorable* - *Intermediate* | 10.5  52.6  36.8 | 6.1  48.8  44.9 | 0.31 |
| *BM Blast (%)* | 51.3 | 50.1 | 0.7 |
| *PB Blast (%)* | 32.5 | 27.9 | 0.26 |
| *WBC, ×10^9^/L* | 24.7 | 26.4 | 0.77 |
| *HGB (g/dL))* | 9.6 | 9.6 | 0.87 |
| *PLT, ×10^7^/L* | 67.3 | 81.4 | 0.22 |
| *Response (%)*   - *Complete response* - *Partial remission* - *Resistance* - *Fail* - *Not treated* | 43.8  1.7  21  7  26.3 | 45.3  1.1  27.3  8.3  17.8 | 0.55 |
| *Relapse (%)*   - *No* - *Yes* | 14  31.5 | 17.1  29.2 | 0.53 |
| *Death (%)*   - *No* - *Yes* | 22.8  77.1 | 16.9  83 | 0.27 |
| *FLT3-ITD (%)*   - *Negative* - *Positive* - *Not detected* | 80.7  14  5.2 | 81.7  16.5  1.7 | 0.21 |
| *FLT3-D835 (%)*   - *Negative* - *Positive* - *Not detected* | 3.5  89.4  5.2 | 5  92.9  1.9 | 0.26 |
| *NPM1 (%)*   - *Wt* - *Mutated* | 59.6  12.2 | 56.8  11.4 | 0.96 |
